# Metabotropic Glutamate Receptor 5 in the Dysgranular Zone of Primary Somatosensory Cortex Mediates Neuropathic Pain in Rats

**DOI:** 10.3390/biomedicines10071633

**Published:** 2022-07-07

**Authors:** Geehoon Chung, Yeong-Chan Yun, Chae Young Kim, Sun Kwang Kim, Sang Jeong Kim

**Affiliations:** 1 Department of Physiology, College of Korean Medicine, Kyung Hee University, 26 Kyungheedae-ro, Dongdaemun-gu, Seoul 02447, Korea; geehoon.chung@khu.ac.kr (G.C.); skkim77@khu.ac.kr (S.K.K.); 2 Department of Physiology, College of Medicine, Seoul National University, 103 Daehak-ro, Jongno-gu, Seoul 03080, Korea; yoon6460@naver.com (Y.-C.Y.); chaeyoung.kim@brain.snu.ac.kr (C.Y.K.); 3 Department of Clinical Korean Medicine, Graduate School, Kyung Hee University, 26 Kyungheedae-ro, Dongdaemun-gu, Seoul 02447, Korea; 4 Department of Biomedical Sciences, College of Medicine, Seoul National University, 103 Daehak-ro, Jongno-gu, Seoul 03080, Korea; 5 Institut du Cerveau—Paris Brain Institute—ICM, INSERM, Sorbonne Université, CNRS, AP-HP, Hôpital de la Pitié Salpêtrière, F-75013 Paris, France

**Keywords:** neuropathic pain, primary somatosensory cortex, dysgranular zone, metabotropic glutamate receptor 5, allodynia, spontaneous pain

## Abstract

The primary somatosensory cortex (S1) plays a key role in the discrimination of somatic sensations. Among subdivisions in S1, the dysgranular zone of rodent S1 (S1DZ) is homologous to Brodmann’s area 3a of primate S1, which is involved in the processing of noxious signals from the body. However, molecular changes in this region and their role in the pathological pain state have never been studied. In this study, we identified molecular alteration of the S1DZ in a rat model of neuropathic pain induced by right L5 spinal nerve ligation (SNL) surgery and investigated its functional role in pain symptoms. Brain images acquired from SNL group and control group in our previous study were analyzed, and behaviors were measured using the von Frey test, acetone test, and conditioned place preference test. We found that metabotropic glutamate receptor 5 (mGluR5) levels were significantly upregulated in the S1DZ contralateral to the nerve injury in the SNL group compared to the sham group. Pharmacological deactivation of mGluR5 in S1DZ ameliorated symptoms of neuropathic allodynia, which was shown by a significant increase in the mechanical paw withdrawal threshold and a decrease in the behavioral response to cold stimuli. We further confirmed that this treatment induced relief from the tonic-aversive state of chronic neuropathic pain, as a place preference memory associated with the treatment-paired chamber was formed in rats with neuropathic pain. Our data provide evidence that mGluR5 in the S1DZ is involved in the manifestation of abnormal pain sensations in the neuropathic pain state.

## 1. Introduction

Neuropathic pain is pathological pain caused by nerve damage [1]. Patients with neuropathic pain experience severe pain in response to a stimulus that does not usually elicit pain, and chronic pain symptoms often persist even in the absence of external stimuli. Even when pain begins with damage to the peripheral nerve, prolonged pain transmission leads to long-term changes in the central nervous system [2,3]. Numerous studies have reported that changes in spinal and supraspinal areas are important in the development and maintenance of neuropathic pain symptoms [1,2,3,4,5,6].

Multiple brain areas are involved in the processing of pain signals at the supraspinal level. The primary somatosensory cortex (S1) is an area where information about bodily sensations is processed and plays a key role in the processing of sensory signals from the body surface and deep tissues [7,8]. S1 processes information related to the location, intensity, and quality of sensory inputs and is capable of discriminating sensations elicited by noxious and non-noxious stimuli. In primates, S1 is subdivided into Brodmann’s areas 1, 2, 3a, and 3b [9,10,11]. Among the subdivisions of primate S1, Brodmann’s area 3a mainly responds to signals of deep somatic input, including noxious sensations [12,13]. In rodents, the dysgranular zone of S1 (S1DZ) is homologous to Brodman’s area 3a in primate S1 [14]. As this area is tightly connected to nociception [15,16], it is expected that changes might occur in this area under chronic pathological pain conditions. However, changes in this area in the prolonged pain state and their functional role in pain symptoms have not yet been studied.

Metabotropic glutamate receptor 5 (mGluR5) is a key mediator of neural plasticity and plays a critical role in changes in the nervous system in various neurological disorders. mGluR5 is highly expressed across the nervous system, including in pain circuits, and is involved in the mechanisms of neuropathic pain [17,18,19,20,21,22,23,24]. In a previous study, we measured mGluR5 availability in the whole rat brain using a positron emission tomography (PET) imaging with an mGluR5-specific radiotracer, [^11^C] ABP688. Animals in the neuropathic pain model showed changes in mGluR5 levels in multiple brain areas. We have previously reported that the upregulation of mGluR5 levels in the medial prefrontal cortex (mPFC) plays a pivotal role in both neuropathic pain and accompanying mood disorders, such as depression and anxiety [24]. In the same study, increased mGluR5 levels were also observed in the S1DZ contralateral to the peripheral nerve injury. Given the sensory-discriminative role of this region, we hypothesized that mGluR5 in the S1DZ plays a role in abnormal sensation in a pathological pain state.

Currently, there are no studies investigating the molecular changes in S1DZ and their functional role in pathological pain. In this study, we investigated whether mGluR5 in the rodent S1DZ plays a role in neuropathic pain symptoms. The relevance of S1DZ-mGluR5 in the manifestation of neuropathic pain symptoms was investigated using various behavioral experiments following microinjection of the mGluR5 antagonist into the S1DZ.

## 2. Materials and Methods

### 2.1. Study Approval

All procedures were approved by the Institutional Animal Care and Use Committee of Seoul National University (SNU-150813-1-1, SNU-161130-4-1) and Kyung Hee University (KHASP-20-104) and were performed according to the Ethical Guidelines of the International Association for the Study of Pain and ARRIVE guidelines.

### 2.2. Animals

Adult male Sprague–Dawley rats (200–250 g) were used in this study. The animals were housed two per cage under constant temperature and humidity conditions with a 12 h light–dark cycle. All animals had free access to food and water. At least one week of acclimation to the animal facilities was provided before beginning the experiments. Animals were randomly assigned to the experimental group, and the experiments were performed in a blinded manner throughout the study. A total of 57 rats were used for behavioral experimental data.

### 2.3. Neuropathic Pain Model

Spinal nerve ligation (SNL) surgery was performed as detailed by Kim and Chung [25]. Under isoflurane anesthesia, a midline incision was made, and the right L6 transverse process was removed using a rongeur. The right L5 spinal nerve was tightly ligated using 5-0 silk and the skin was sutured. In sham surgery, the rats underwent the same procedure except for ligation. After surgery, animals were returned to their home cages and monitored during recovery.

### 2.4. Cannula Implantation and Microinjection

Cannulation surgery was performed in the SNL group 9 days after SNL surgery. The head of each animal was fixed in a stereotaxic frame under deep isoflurane anesthesia. A hole was made in the skull with a mini-drill, and a guide cannula was implanted with dental cement and mini-screws. The coordinates of AP −0.2 mm, ML −4.6 mm (left), and DV −2.9 mm from the bregma were targeted, which placed the tip of the guide cannula 0.5 mm above the S1DZ injection site. A dummy cannula was used to prevent the tissue from entering the bottom of the guide cannula. A recovery period of at least 6 days was provided. Microinjection of the drug into the S1DZ was conducted 20 days after SNL surgery using an internal cannula with 0.5 mm additional length beyond the guide cannula. The internal cannula was inserted into the guide cannula after the removal of the dummy cannula, and 0.5 μL of the solution was injected into the S1DZ through the internal cannula. The injection site was verified using dye injection after the completion of the experiment.

### 2.5. Von Frey Test

To assess neuropathic mechanical allodynia, the paw withdrawal threshold of the animal was measured using the von Frey test with the up-down method [26]. The animals were placed on a grid mesh floor under a transparent acrylic cage and allowed to acclimatize for 20 min. Von Frey filaments (3.61, 3.84, 4.08, 4.31, 4.56, 4.74, 4.93, and 5.18) were applied to the right hind paw and behavioral responses were observed. The 50% paw withdrawal threshold was calculated as described by Chaplan [27]. A value of 15 g was used as the cut-off. Rats with a hypersensitive paw at baseline (paw withdrawal threshold < 10 g) were excluded and not subjected to surgery.

### 2.6. Acetone Test

The acetone test was used to assess the neuropathic cold allodynia. Under testing conditions similar to the von Frey test, 50 μL of acetone was applied to the test area of the right hind paw and the behavioral responses were monitored and scored (0: no response; 1: quick withdrawal or flicking; 2: prolonged withdrawal or repeated flicking; 3: repeated flicking of the paw with licking) [28]. Acetone application was repeated three times, and the average value was then calculated.

### 2.7. Conditioned Place Preference (CPP) Test

The CPP test was used to evaluate the effect of the S1DZ-mGluR5 blockade on the tonic-aversive state of neuropathic animals [29]. In this test paradigm, the animal was first acclimatized to conditioning chambers (with vertical or horizontal visual cues) connected to a neutral chamber for 30 min. The next day, the animal was introduced again to the three chambers for 15 min, and the preconditioning preference was recorded. Animals that spent less than 20% of their time in any one chamber were excluded. On the morning of the next day, the animal was microinjected with vehicle solution in the S1DZ and conditioned in a randomly chosen chamber (chamber 1) for 30 min. During this conditioning period, the animal was not allowed to enter other chambers. In the afternoon, the animal was again microinjected with 3-((2-Methyl-1,3-thiazol-4-yl)ethynyl)pyridine hydrochloride (MTEP) into the S1DZ and conditioned to a counterbalanced chamber (chamber 2). The next day, the animal was placed in the CPP chambers with free access to all chambers. Animal behavior was video-recorded for 15 min. The CPP score was calculated as the time spent in the MTEP-conditioned chamber (chamber 2) divided by the total time spent in the MTEP-conditioned chamber (chamber 2) and the vehicle-conditioned chamber (chamber 1).

### 2.8. PET Analysis

mGluR5 availability levels were measured using PET imaging data acquired in our previous study [24]. Briefly, the SNL group and sham group rats underwent PET scans with a mGluR5-specific radiotracer, [^11^C] ABP688, using a microPET scanner (eXplore VISTA, GE Healthcare, Waukesha, Wisconsin, USA) at 16–25 days after surgery. The list-mode data for 60 min were acquired and reconstructed into a single static image and multiple dynamic frames. mGluR5 availability was measured by calculating the non-displaceable binding potential (BP_ND_) of [^11^C] ABP688 using a simplified reference tissue model with the cerebellum as a reference region. All images were co-registered, normalized, resampled, smoothed, and analyzed using SPM8 and SPM12 software. A voxel-by-voxel two-sample *t*-test was performed and the statistical map was overlaid on the magnetic resonance imaging (MRI) template [30] for visualization. To compare mGluR5 availability in the S1DZ in this study, a 0.5 mm radius spherical region-of-interest (ROI) was defined using the MarsBaR toolbox [31], at the location of S1DZ with the following coordinates: ML ±4.6 mm, AP −0.2 mm and DV −3.4 mm from the bregma. Proportionally scaled values were extracted for each subject.

### 2.9. Drugs

MTEP was used to block mGluR5 activity. MTEP was dissolved in distilled water (100 mM) and diluted to the final concentration with artificial cerebrospinal fluid before microinjection. MTEP (10 nmol) was microinjected into the S1DZ.

### 2.10. Statistical Analysis

Whole-brain analysis of the PET images was performed using a voxel-by-voxel two-sample *t*-test. ROI comparisons were performed using two-sample *t*-tests. Paw withdrawal thresholds were analyzed using a two-way repeated-measures analysis of variance (ANOVA). The acetone test scores were analyzed using a two-sample *t*-test. For CPP experiments, the time difference between chambers was compared between chamber 1 (vehicle-conditioned chamber) and chamber 2 (MTEP-conditioned chamber) using a two-sample *t*-test for each group, and the preference for the MTEP-conditioned chamber was compared between the pre- and post-conditioning periods in each group using a paired *t*-test. Two-tailed tests were used for all analyses. Data are presented as mean ± SEM.

## 3. Results

### 3.1. mGluR5 Availability Was Upregulated in the Left S1DZ of Animals with Right SNL Surgery

**Figure 1:**
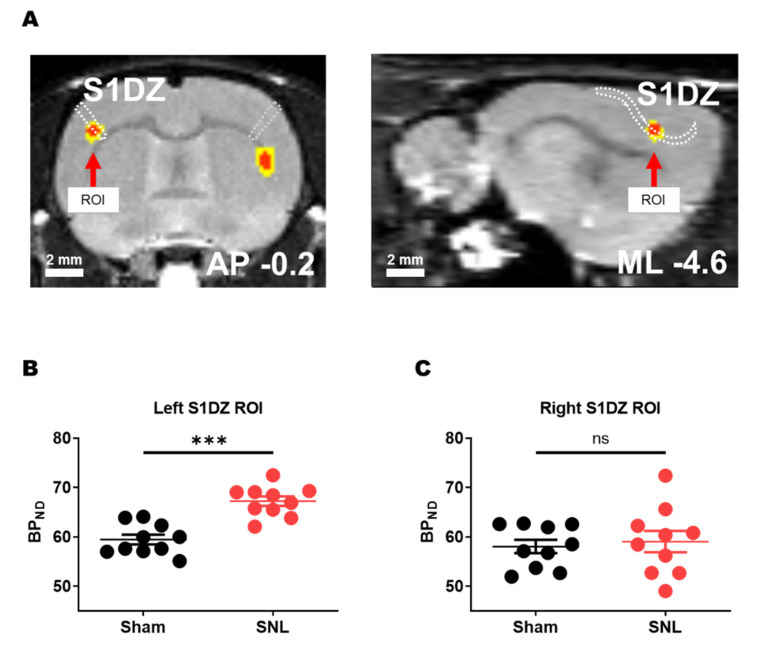
The SNL group showed an increased mGluR5 level in the S1DZ contralateral to the peripheral nerve injury. (**A**) Result images of a voxel-by-voxel two-sample *t*-test comparison between PET images of the SNL and sham group (SNL > sham). Significance maps of uncorrected *p* < 0.001 (red) or *p* < 0.005 (yellow) overlaid on a single MRI template are shown with coronal and sagittal section images of appropriate coordinates. The S1DZ areas are drawn with a white dotted line. (**B**) mGluR5 availability levels in the left S1DZ ROI were higher in rats subjected to right L5 SNL surgery compared with that in sham group rats (*** *p* < 0.001; two-sample *t*-test; sham = 59.45 ± 0.99, *n* = 10; SNL = 67.22 ± 0.96, *n* = 10). (**C**) mGluR5 levels in the right S1DZ ROI did not differ between groups (ns: not significant; two-sample *t*-test; sham = 58.03 ± 1.36, *n* = 10; SNL = 59.03 ± 2.18, *n* = 10).

In a previous PET study, we reported that multiple brain regions showed changes in mGluR5 levels in animals with neuropathic pain subjected to right L5 SNL surgery [24]. Among these regions, the left S1DZ showed a prominent increase in mGluR5 availability compared to that in the sham group. The peak voxel was located at the coordinates of ML −4.6 mm, AP −0.2 mm, and DV −3.4 mm from the bregma (Figure 1A). In this study, we compared individual mGluR5 levels between the SNL and sham group using a 0.5 mm radius spherical ROI at this location. We confirmed that the mGluR5 levels in the left S1DZ ROI of the SNL group were significantly higher than those in the sham group (Figure 1B). In comparison, using flipped coordinates (ML +4.6 mm) mGluR5 levels in the right S1DZ ROI did not differ between groups (Figure 1C).

### 3.2. Pharmacological Blockade of mGluR5 in the S1DZ Ameliorated Mechanical Allodynia in Animals with Neuropathic Pain

**Figure 2:**
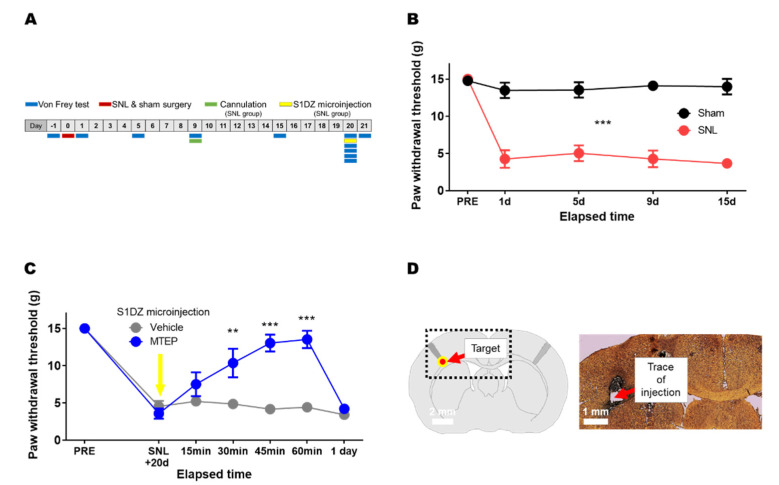
Microinjection of the mGluR5 antagonist into the S1DZ ameliorated SNL-induced mechanical allodynia. (**A**) Experimental design. (**B**) SNL group showed a reduction in the paw withdrawal threshold in the von Frey test, indicating mechanical allodynia (*** *p* < 0.001; two-way repeated-measures ANOVA; sham *n* = 12, SNL *n* = 11). (**C**) Microinjection of mGluR5 antagonist MTEP into the S1DZ ameliorated neuropathic mechanical allodynia symptom of SNL group (** *p* < 0.01, *** *p* < 0.001; two-way repeated-measures ANOVA with Bonferroni’s test; vehicle *n* = 5, MTEP *n* = 6). (**D**) Verification of cannula trajectory.

To identify the functional role of mGluR5 in S1DZ, we examined the effect of microinjection of MTEP, a highly specific noncompetitive mGluR5 antagonist with inverse agonist activity, into the S1DZ. Animals were subjected to either SNL or sham surgery, and the withdrawal threshold of the affected hind paw was measured repeatedly using the von Frey test. A detailed explanation is provided in the Materials and Methods section, and the schedule of behavioral testing, surgery, cannulation, and microinjection is summarized in the experimental design (Figure 2A). We first confirmed the successful induction of neuropathic pain in the SNL group. As shown by the reduction in the paw withdrawal threshold, mechanical allodynia, a representative symptom of neuropathic pain, was seen in the right hind paw and stably maintained across the testing period (Figure 2B). We then infused MTEP into the left S1DZ of SNL rats through the implanted cannula and examined the changes in symptoms. The microinjection of MTEP into S1DZ induced a potent anti-allodynic effect, as shown by a significant increase in the paw withdrawal threshold (Figure 2C). The paw withdrawal threshold gradually increased over at least 60 min to reach the pre-SNL level, and this analgesic effect disappeared within the next 24 h. Control SNL rats microinjected with vehicle showed no change in the paw withdrawal threshold. The microinjection site was verified at the end of the experiment (Figure 2D).

### 3.3. Pharmacological Blockade of mGluR5 in the S1DZ Ameliorated Cold Allodynia in Neuropathic Pain Animals

**Figure 3:**
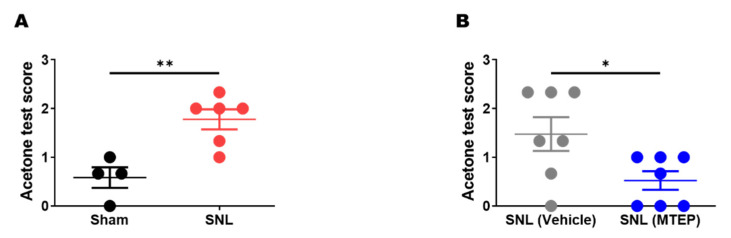
Microinjection of the mGluR5 antagonist into the S1DZ ameliorated SNL-induced cold allodynia. (**A**) SNL group showed increased behavioral responsiveness in the acetone test, indicating cold allodynia (** *p* < 0.01; two-sample *t*-test; sham = 0.58 ± 0.20, *n* = 4; SNL = 1.78 ± 0.20, *n* = 6). (**B**) Microinjection of mGluR5 antagonist MTEP into the S1DZ ameliorated cold allodynia symptom of SNL group (* *p* < 0.05; two-sample *t*-test; Vehicle = 1.48 ± 0.35, *n* = 7; MTEP = 0.52 ± 0.19, *n* = 7).

In the next experiment, we investigated whether MTEP microinjection into the S1DZ could ameliorate cold allodynia symptoms in SNL group animals. Consistent with previous studies, neuropathic animals showed enhanced nocifensive behaviors to the plantar application of acetone, representing symptoms of cold allodynia (Figure 3A). In a new batch of experiments, SNL rats were cannulated for S1DZ microinjection and then assigned to either the vehicle or MTEP administration group. The acetone test was performed 30 min after MTEP microinjection into the S1DZ. We found that MTEP-treated SNL rats showed significantly fewer behavioral responses to acetone application than vehicle-treated SNL rats did (Figure 3B).

### 3.4. Animals with Neuropathic Pain Showed a Preference for the Chamber Conditioned with Pharmacological Blockade of mGluR5 in the S1DZ

**Figure 4:**
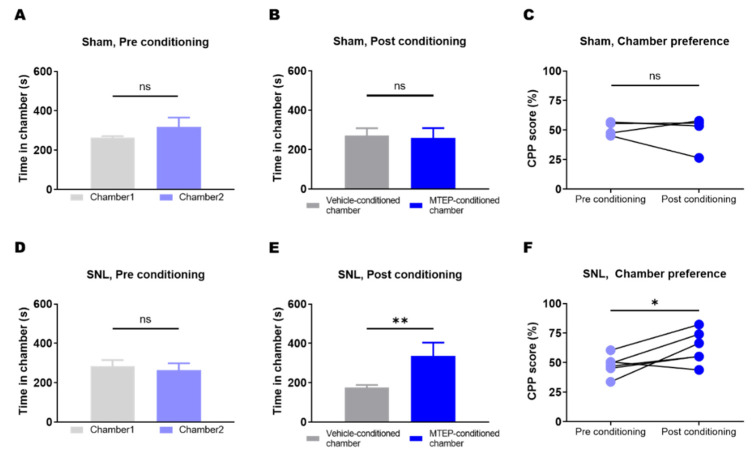
Microinjection of the mGluR5 antagonist into the S1DZ ameliorated SNL-induced spontaneous ongoing pain. (**A**) Baseline chamber preference was absent in the sham group (ns: not significant; two-sample *t*-test; *n* = 4). (**B**) Even after conditioning, time spent in each chamber was not different in the sham group (ns: not significant; two-sample *t*-test; *n* = 4). (**C**) The preference for the chamber did not change before and after the MTEP conditioning in the sham group (ns: not significant; paired *t*-test; *n* = 4). (**D**) Baseline chamber preference was absent in the SNL group (ns: not significant; two-sample *t*-test; *n* = 6). (**E**) After the conditioning, the SNL group spent more time in the MTEP-conditioned chamber than in the vehicle-conditioned chamber (** *p* < 0.01; two-sample *t*-test; *n* = 6). (**F**) The SNL group developed a preference for a chamber conditioned with MTEP microinjection into the S1DZ (* *p* < 0.05; paired *t*-test; *n* = 6).

We further investigated whether MTEP microinjection into the S1DZ affected spontaneous ongoing pain in the SNL group. The CPP test, an analysis that can be used to measure the effectiveness of the intervention on tonic-aversive pain perception, was performed according to previous studies [22,24,29,32]. Before the conditioning process, animals in both groups showed no preference for either chamber (Figure 4A,D). After conditioning, SNL animals, but not sham animals, spent significantly more time in the chamber in which they were conditioned with MTEP microinjection into the S1DZ (Figure 4B,E). This preference indicates that SNL animals perceived the microinjection of MTEP into the S1DZ as a reward. We inferred that the relief from the spontaneous ongoing pain state would act as a motivational factor that induces chamber preference, as no external stimuli were given during the experiments and only animals with neuropathic pain (SNL group) showed chamber preference.

## 4. Discussion

Taken together, our data demonstrated the critical involvement of S1DZ-mGluR5 in neuropathic pain symptoms. Previously, we showed changes in mGluR5 availability in the brains of animals with neuropathic pain [24]. In that study, we performed experiments focusing on an ROI located in the prelimbic cortex, a subregion of rodent mPFC. The SNL rats demonstrated increased mGluR5 level within this region, and pharmacological blockade of it induced a potent analgesic effect and anti-depressive effect. Conversely, naïve rats developed neuropathic pain-like symptoms and negative mood symptoms such as depression and anxiety after viral overexpression of mGluR5 in this region, consistent with the well-known role of the prelimbic cortex in emotional and affective processing. The current study examined the functional role of the somatosensory area, which plays a critical role in processing sensory information. We focused on an ROI located in the left S1DZ of animals subjected to right SNL surgery. mGluR5 levels were upregulated in the S1DZ contralateral to the peripheral nerve injury, and deactivation of the receptor ameliorated the symptoms of neuropathic pain. This indicates that the nociplastic change in this brain region is critically involved in the manifestation of neuropathic pain symptoms.

The somatosensory cortices are located in the parietal lobe and play a pivotal role in discriminating sensory modalities and localization of sensations [7,8]. Although controversial [33], somatosensory cortices are known to be involved in the development and maintenance of chronic pain symptoms [34]. Multiple studies have reported changes in the somatosensory cortex following nerve injury [35,36,37,38]. Animal studies have shown that pain induced by peripheral nerve injury alters neuronal activity and glial activation in the affected S1 region, leading to abnormal pain processing in the brain [3,34]. Conversely, mimicking these changes in S1 results in neuropathic pain-like symptoms [3,23]. Accumulating evidence has shown an important role for S1 reorganization in a prolonged pain state.

Among the somatosensory cortices, Brodmann area 3a in primates receives input from sensory receptors and nociceptors of cutaneous skin and deep tissues to process the information on its own somatotopic map [7,12]. In rodents, the dysgranular zone (also known as the transitional zone) of S1 is considered homologous to primate area 3a. As in the case of the Brodmann area 3a, rodent S1DZ is distinct from other S1 regions in terms of its anatomical location, cellular characteristics, and functional role in sensory perception. S1DZ can be distinguished from the other cortices by its thinning in layer 4 and is positioned as if encapsulating other S1 regions. Recent studies have reported increased activity of S1DZ neurons in response to noxious stimuli [14,15], implying a role for this region in pain perception. However, changes in S1DZ in pathological pain conditions have not been studied despite the involvement of this region in noxious sensations. To the best of our knowledge, this is the first study to report the functional role of S1DZ in neuropathic pain.

In terms of the mechanisms of neuropathic pain, mGluR5 plays a bidirectional role in pain processing, depending on the pain pathway in which mGluR5 acts. In the peripheral nerve and spinal cord, mGluR5 plays a role in pain transmission [20,39]. Injection of various mGluR5 antagonists induced analgesic effects in neuropathic pain animals [20,40]. In the brain, mGluR5 in the cortical regions mainly promotes pain perception [23,39], whereas mGluR5 in the pain modulatory area, such as periaqueductal gray, inhibits pain [22,41,42]. The potent anti-allodynic effect induced by microinjection of MTEP into the S1DZ in the current study indicates that mGluR5 in this region is critically involved in the distorted sensory-discriminative function. We infer that the upregulation of S1DZ-mGluR5 in a neuropathic pain state perturbs sensory discrimination, leading to the perception of innocuous stimuli as noxious sensations. That is, increased mGluR5 in S1DZ facilitates the manifestation of pain symptoms. Intervention with MTEP treatment successfully ameliorated the symptoms of mechanical and cold allodynia, supporting the idea above.

Despite the well-established role of S1 in sensory processing, one might ask if the reduction in pain behavior we reported is caused by motor deficit rather than pain relief. Indeed, previous studies have reported that motor deficits were observed after S1 lesions [43]. However, the study suggested that the deficits may not be due to difficulty with executing motor commands. Rather, the authors related disrupted learning of new motor tasks to motor dysfunction, as deficits were attenuated if the task had been learned before the S1 lesion. We suggest that the reduction in pain behavior induced by the S1DZ-mGluR5 blockade cannot be attributed to motor deficits. We measured the paw withdrawal threshold repeatedly before and after the SNL surgery, and this behavioral response is not newly learned after the MTEP injection into the S1DZ. In addition, we performed CPP experiments that measure the preference of the subject for the treatment. If the reduction in pain behavior was caused by the motor deficit rather than pain relief, the treatment (i.e., blockade of mGluR5 in the S1DZ) would not be able to induce chamber preference in SNL rats. In our experiments, however, the SNL rats preferred the treatment-paired chamber, which supports that the altered behavioral response was related to pain relief.

The results of CPP experiments also suggest that mGluR5 changes in the S1DZ might be associated with spontaneous ongoing pain symptoms in neuropathic pain conditions. Only the SNL group, but not the sham group, developed a preference for a chamber conditioned with pharmacological blockade of S1DZ-mGluR5, implying that the increased mGluR5 levels in the neuropathic pain state are closely related to motivation (i.e., pain and relief). As a molecular modulator of glutamate receptors, mGluR5 activation in the nervous system mainly enhances neuronal activity and thereby increases pain perception [39,44]. As such, upregulated mGluR5 in S1DZ may enhance excitatory glutamatergic transmission and intrinsic excitability of neurons, increasing the chance of firing action potentials. Previous studies have reported that mGluR5 promotes spontaneous neuronal activity and is often persistently active [22,45,46,47]. This raises the possibility that the mGluR5 upregulation observed in the pain pathway might be a mechanism of spontaneous ongoing pain. Spontaneous neuronal firing associated with increased mGluR5 in the brain cortex may be a mechanism that helps to explain the maintenance of persistent ongoing pain. Further studies are needed to clarify this issue, as the electrophysiological properties of S1DZ neurons and their changes under pathological pain conditions are currently unknown.

The main limitation of the current study was that we did not specify the neural circuits within S1DZ in which mGluR5 is altered. Future studies need to investigate in which cell types, cellular location, and cortical layers mGluR5 is altered. More research on mechanisms of molecular change in this area is also needed to reveal the therapeutic targets.

## 5. Conclusions

In conclusion, our data provide evidence that mGluR5 in the S1DZ is involved in the abnormal sensory perception in the neuropathic pain state. Among the alteration of mGluR5 levels in the various brain regions demonstrated in our previous study, this work highlights an increased mGluR5 level in the S1DZ contralateral to peripheral nerve injury. Considering homology between human and rodent S1, blockade of mGluR5 in human Brodmann area 3a may present a novel strategy for the management of chronic neuropathic pain.

## Data Availability

Further information and requests for data used during the current study should be directed to and will be fulfilled by the corresponding author.
